# Dexmedetomidine Attenuates Lipopolysaccharide-Induced Sympathetic Activation and Sepsis via Suppressing Superoxide Signaling in Paraventricular Nucleus

**DOI:** 10.3390/antiox11122395

**Published:** 2022-12-02

**Authors:** Jin-Hua Bo, Jing-Xiao Wang, Xiao-Li Wang, Yang Jiao, Ming Jiang, Jun-Liu Chen, Wen-Yuan Hao, Qi Chen, Yue-Hua Li, Zheng-Liang Ma, Guo-Qing Zhu

**Affiliations:** 1 Key Laboratory of Targeted Intervention of Cardiovascular Disease, Collaborative Innovation Center for Cardiovascular Disease Translational Medicine, Department of Physiology, Nanjing Medical University, Nanjing 211166, China; 2 Department of Anesthesiology, The Affiliated Drum Tower Hospital, Medical School of Nanjing University, Nanjing 210008, China; 3 Department of Pathophysiology, Nanjing Medical University, Nanjing 211166, China

**Keywords:** dexmedetomidine, sepsis, sympathetic activity, paraventricular nucleus, inflammation, oxidative stress, cAMP, gamma-aminobutyric acid (GABA)

## Abstract

Sympathetic overactivity contributes to the pathogenesis of sepsis. The selective α2-adrenergic receptor agonist dexmedetomidine (DEX) is widely used for perioperative sedation and analgesia. We aimed to determine the central roles and mechanisms of DEX in attenuating sympathetic activity and inflammation in sepsis. Sepsis was induced by a single intraperitoneal injection of lipopolysaccharide (LPS) in rats. Effects of DEX were investigated 24 h after injection of LPS. Bilateral microinjection of DEX in the paraventricular nucleus (PVN) attenuated LPS-induced sympathetic overactivity, which was attenuated by the superoxide dismutase inhibitor DETC, cAMP analog db-cAMP or GABA_A_ receptor antagonist gabazine. Superoxide scavenger tempol, NADPH oxidase inhibitor apocynin, adenylate cyclase inhibitor SQ22536 or PKA inhibitor Rp-cAMP caused similar effects to DEX in attenuating LPS-induced sympathetic activation. DEX inhibited LPS-induced superoxide and cAMP production, as well as NADPH oxidase, adenylate cyclase and PKA activation. The roles of DEX in reducing superoxide production and NADPH oxidase activation were attenuated by db-cAMP or gabazine. Intravenous infusion of DEX inhibited LPS-induced sympathetic overactivity, NOX activation, superoxide production, TNF-α and IL-1β upregulation in the PVN and plasma, as well as lung and renal injury, which were attenuated by the PVN microinjection of yohimbine and DETC. We conclude that activation of α2-adrenergic receptors with DEX in the PVN attenuated LPS-induced sympathetic overactivity by reducing NADPH oxidase-dependent superoxide production via both inhibiting adenylate cyclase-cAMP-PKA signaling and activating GABA_A_ receptors. The inhibition of NADPH oxidase-dependent superoxide production in the PVN partially contributes to the roles of intravenous infusion of DEX in attenuating LPS-induced sympathetic activation, oxidative stress and inflammation.

## 1. Introduction

Sepsis is closely associated with excessive persistent inflammation and organ dysfunction [1]. Norepinephrine is the primary neurotransmitter released by sympathetic nerve terminals [2] and it plays a role in regulating the immune response [3]. Sympathetic overactivity and increased plasma norepinephrine level were observed in septic patients and experimental septic animals [4]. Intraportal injection of norepinephrine at a concentration under septic conditions increased circulating interleukin (IL)-1β and tumor necrosis factor (TNF)-α, similar to that found in sepsis [5]. Norepinephrine-induced TNF-α and IL-1β production was mediated by α2-adrenergic receptors (α2Rs) [6]. Sympathetic overactivity and increased norepinephrine level in sepsis contribute to inflammation and dysfunction of multiple organs such as cardiac dysfunction and renal injury [7,8,9,10]. Excessive sympathetic activity not only promotes vasoconstriction, but also causes norepinephrine-induced extracellular vesicle release from adventitial fibroblasts of arteries [2]. Extracellular vesicle-mediated angiotensin-converting enzyme transfer further contributes to changes in vascular function and structure [11,12]. Central sympatholytics attenuated inflammation and prolonged survival in a mouse model of caecal ligation and puncture-induced sepsis, and excessive sympathetic activation is crucial in the pathogenesis of sepsis [13].

Paraventricular nucleus (PVN) of the hypothalamus in the brain is important in regulating autonomic nerve activity and cardiovascular function [14]. Presympathetic neurons in the PVN are the main source of excitatory drive for sympathetic activity [15]. A variety of neurotransmitters and signal molecules contribute to the modulation of sympathetic outflow, mainly including glutamate, gamma-aminobutyric acid (GABA), angiotensin II, norepinephrine, reactive oxygen species (ROS) and inflammatory cytokines [16]. Catecholaminergic inputs within the PVN primarily release NE, which acts on one or more adrenergic receptors including α1R, α2R and β2R. Parvocellular neurons of the PVN exposed to norepinephrine exhibit excitatory effects via α1R, or inhibitory effects via α2R [17]. α2R in the PVN tonically restrains norepinephrine synthesis, release and turnover in presympathetic neurons of the PVN, and limits immobilization-induced peripheral sympathetic activation in conscious rats [18]. α2R is subdivided into α2A, α2B and α2C subtypes, and is expressed in brain and peripheral tissues [19]. Sympathetic outflow is mainly regulated by activation of the presynaptic α2A subtype, which is the predominant α2R subtype in the brain and exerts an inhibitory role in sympathetic activity [20]. α2R agonist clonidine serves as the first-generation central antihypertensive drug that reduces sympathetic outflow [21].

The α2R agonist dexmedetomidine (DEX) is becoming increasingly popular in the intensive care management of patients due to its sedative, analgesic, sympatholytic and antianxiety properties, with fewer adverse side effects than clonidine [22]. DEX shows anti-inflammatory effects through its action on α2R and plays beneficial roles in organ protection during sepsis [23]. Excessive persistent sympathetic activity occurs in septic patients and animal models [4], while DEX inhibits sympathetic activity and inflammation in sepsis [22,23]. The α2R is widely distributed in the brain to regulate various functions including autonomic control, and abundant α2R is found in the hypothalamic PVN [24]. DEX can easily cross the blood-brain barrier [25] and can be administered by intravenous injection, intramuscular injection, nasal drip, buccal mucosa or orally [26]. However, little is known about the central roles and mechanisms of DEX in attenuating sympathetic overactivity, oxidative stress, inflammation and organ injury in sepsis. We hypothesized that the beneficial roles of systemic administration of DEX may be at least partially mediated by its central effects on the control of sympathetic outflow. We aimed to determine the roles and underlying mechanisms of DEX in the PVN in inhibiting sympathetic outflow, and to determine whether the central sympatholytic effects of DEX contribute to its beneficial effects in attenuating oxidative stress, inflammation and organ injury in lipopolysaccharide (LPS)-induced sepsis. We aimed to determine the central roles and mechanisms of DEX in attenuating sympathetic activity and inflammation in sepsis.

## 2. Materials and Methods

### 2.1. Rats

Male Sprague–Dawley rats weighting between 300 and 350 g were purchased from Jiangsu Laboratory Animal Center (Nanjing, China) and housed under controlled humidity, temperature and lighting (12 h light, 12 h dark). The rats were given free access to standard rodent chow and tap water. Studies conformed to the Guide for the Care and Use of Laboratory Animals (8th edn, NIH). Experiments were approved by the Experimental Animal Care and Use Committee of Nanjing Medical University (IACUC2107007 & 2206033, Nanjing, China).

### 2.2. Septic Animal Model

Intravenous injection of LPS (3 mg/Kg) was used to induce sepsis [27,28,29]. LPS was dissolved in saline, and the control rats received the same volume of saline intravenously. Effects of DEX were investigated 24 h after intravenous injection of saline or LPS. Renal sympathetic nerve activity (RSNA) was enhanced, plasma norepinephrine, TNF-α and IL-1β levels were increased in LPS-treated rats.

### 2.3. General Procedures

Rats were anaesthetized by intraperitoneal injection of a mixture of urethane (800 mg/Kg) and α-chloralose (40 mg/Kg). A vertical incision in the middle of the neck was performed. Positive pressure ventilation was carried out with a Model 683 ventilator (Harvard Apparatus Inc., Holliston, MA, USA) via tracheal intubation. Left kidney and renal nerves were exposed via a left flank incision for RSNA recording. Mean arterial pressure (MAP), heart rate (HR) and RSNA were recorded with a data acquisition system (8SP, ADInstruments, Bella Vista, NSW, Australia). Rats were allowed to stabilize for at least 30 min before intervention. Finally, rats were euthanized with pentobarbital sodium (100 mg/Kg, ip). A total of 194 rats were used in this study, in which 8 rats were excluded owing to the inaccurate PVN microinjection sites (*n* = 6), and lower blood pressure or severe bleeding during surgery (*n* = 2).

### 2.4. RSNA Recording

A left flank incision was made and the left renal nerve was separated. The distal end of the nerve was severed to eliminate the nerve activity from the kidney. A pair of platinum electrodes were placed under the central end of the nerve and then immersed in warm mineral oil (37 °C). The RSNA was amplified 1000 times with a 100–3000 Hz bandpass filtration with a differential amplifier (DP-304, Warner Instruments, Hamden, CT, USA). The signals were integrated using LabChart 8 software (ADInstruments, Bella Vista, NSW, Australia) at 100 ms time constant. At the end of the recording, the nerve was cut at its central end to obtain the background noise. The RSNA value was equal to the raw RSNA minus the background noise [30].

### 2.5. PVN Microinjection

A stereotaxic frame (Stoelting, Chicago, IL, USA) was used to fix the rat in a prone position. A midline incision was made to expose the skull. Microinjection sites were determined according to Paxinos and Watson’s rat atlas as we previously reported [31], which is 0.4 mm lateral to the midline, 1.8 mm caudal from bregma, and 7.9 mm ventral to the dorsal surface. Bilateral PVN microinjections were completed within 1 min. The volume of microinjection was 50 nL for each microinjection site. After the experiment, Evans blue was injected the same as the PVN microinjection and histological verification was performed under a microscope.

### 2.6. Heart Rate Variability

Heart rate variability was used to assess sympathetic and parasympathetic activity levels as we previously reported [32]. Briefly, electrocardiograph (ECG) was recorded at the standard II configuration via subcutaneous electrodes with the PowerLab system and its software for heart rate variability analysis in the frequency domain. Low-frequency (LF) stands for both sympathetic and parasympathetic activities, high-frequency (HF) reflects parasympathetic activity, and the LF/HF ratio indicates sympathetic activity. We evaluated the power of LF, HF, very low frequency power (VLF) and total power (TP) at 0.20 to 0.75 Hz, 0.75 to 2.50 Hz, 0 to 0.20 Hz and 0 to 3.00 Hz, respectively. The normalization (norm) values of these indices better reflect sympathetic–parasympathetic activity and are expressed as normalized units (nu). The norm LF is an index of sympathetic activity, which is calculated as norm LF = 100 × LF/(TP-VLF). The norm HF serves as an index of parasympathetic activity, which is calculated as norm HF = 100 × HF/(TP-VLF). The norm LF/norm HF ratio reflects the balance between sympathetic activity and parasympathetic activity.

### 2.7. PVN Preparation for Measurements

Coronal sections with a thickness of 450 µm were prepared at the PVN level of the brain using a cryostat microtome (Model CM1900, Leica, Wetzlar, Germany). A 15-gauge needle was used to punch the PVN out from the slices. Then, the tissue was homogenized and centrifuged in a lysis buffer for a variety of measurements. Total protein content in the tissue was measured with a Bradford assay kit (BCA; Pierce, Santa Cruz, CA, USA).

### 2.8. Measurement of Superoxide Level and NADPH Oxidase Activity

Lucigenin-derived chemiluminescence method was used to determine NADPH oxidase activity and superoxide production. Photon emission was initiated by adding both NADPH (100 μM) and dark-adapted lucigenin (5 μM) for the measurement of NADPH oxidase activity. Photon emission was initiated by adding dark-adapted lucigenin (5 μM) for examining superoxide production. The average light emission was obtained by measuring 10 times in 10 min with a luminometer (Model 20/20n, Turner, CA, USA). The background chemiluminescence of the buffer containing lucigenin (5 μM) was measured. Values were expressed in relative mean light unit (MLU)/min/mg protein [33].

### 2.9. Detection of Superoxide Production in Brain Slice

Superoxide production was detected with a specific dihydroethidium (DHE) fluorogenic probe as we reported previously [34]. Slices from different groups were processed in parallel. All settings of the detector and laser were left unchanged during the experiments. The DHE fluorescence was detected by a fluorescence microscope (DP70, Olympus Optical, Tokyo, Japan).

### 2.10. Measurements with Commercial Kits

TNF-α and IL-1β levels were measured with their commercial ELISA kits (Uscn Life Science Inc., Houston, TX, USA), respectively. Norepinephrine levels were determined with the ELISA kits from R&D systems (Minneapolis, MN, USA). Adenylate cyclase activity was detected by an adenylate cyclase activity assay kit (mlbio co., Shanghai, China). The cAMP levels were determined with a cyclic adenosine monophosphate assay kit (Nanjing Jiancheng Bioengineering Institute, Nanjing, Jiangsu, China). Protein kinase A (PKA) activity was detected by a PKA activity kit (Enzo Life Sciences, Ann Arbor, MI, USA).

### 2.11. Quantitative RT-PCR

Total RNA was extracted from PVN samples with Trizol reagent (Life Technologies, Gaithersburg, MD, USA). A PrimeScript RT reagent kit (Vazyme Biotech Co., Ltd., Nanjing, Jiangsu, China) was used for reverse transcription. qRT-PCR was performed with SYBR Green Master Mix (Vazyme Biotech Co., Ltd., Nanjing, Jiangsu, China) on the StepOnePlus™ Real-Time PCR System (Applied Biosystems, Foster City, CA, USA). The data were analyzed with the Ct (2-ΔΔCt) comparison method. GAPDH was used as a normalized control. Primers were purchased from Invitrogen Corporation (Shanghai, China) and are listed in the online supplementary table (Table S1).

### 2.12. Western Blot

The PVN sample was homogenized in the lysis buffer. The total protein in supernatant was measured with BCA protein assay kits (Thermo Fisher Scientific, Rockford, IL, USA). The same amount of total protein was separated by 10% SDS-PAGE and then transferred to PVDF membrane. Antibodies against α2A receptor protein were obtained from ABclonal Technology Co (Wuhan, Hubei, China). β-actin was used as a normalized control. Protein bands were detected with an enhanced chemiluminescence detection kit (Thermo Fisher Scientific, Rockford, IL, USA). All antibodies used in the present study were derived from rabbits.

### 2.13. Hematoxylin and Eosin Staining

Lungs and kidneys were fixed and preserved with 4% paraformaldehyde for embedding, sectioning, HE staining and analyses. The sections were observed and photographed under a light microscope.

### 2.14. Immunofluorescence Staining

Samples were fixed in paraformaldehyde at 4 °C for 2–4 h, cryoprotected overnight in PBS containing 20–30% sucrose and sectioned with a cryotome at a 20 μm thickness. The plates were rinsed with PBS three times and then treated with 0.2% Triton X-100 and 3% BSA for 2 h at room temperature. The sections were incubated with GABA_A_R antibody (1:500) and α2AR antibody (1:200) overnight at 4 °C. Nuclei of cells were stained with 4′,6-diamino-2-phenylindole (DAPI). Sections were incubated with two different fluorescein labeled secondary antibodies at room temperature for 1 h. Immunofluorescence was detected with a confocal laser scanning microscope (Bio-Rad, Hercules, CA, USA). GABA_A_R antibody was purchased from Synaptic Systems (Göttingen, Germany), and α2AR antibody was purchased from ABclonal (Wuhan, Hubei, China).

### 2.15. Chemicals

Tempol, apocynin, db-cAMP, SQ22536, vigabatrin, gabazine, yohimbine and clonidine were obtained from MedChemExpress (Monmouth Junction, NJ, USA). LPS, CGP35348, DEX and DETC were purchased from Sigma (St Louis, MO, USA). Rp-cAMP was purchased from Beyotime (Shanghai, China). Apocynin and CGP35348 were dissolved in PBS containing 1% DMSO and the vehicle was used as a control. Other chemicals were dissolved in PBS. PBS was used as a control.

### 2.16. Statistical Analysis

Experiments were performed in a randomized and double-blinded mode. Data are expressed as mean ± SE. Power analysis was used to determine the number of animals or replicates for the power of more than 0.8 significant difference. The differences between the two groups were compared with nonpaired Student’s t-tests. The differences among groups were compared with one-way or two-way ANOVA followed by a Student–Newman–Keuls (SNK) test. Time effects of LPS or DEX were analyzed with repeated measures ANOVA. *p* < 0.05 was considered significant.

## 3. Results

### 3.1. LPS-Induced Sympathetic Activation

**Figure 1:**
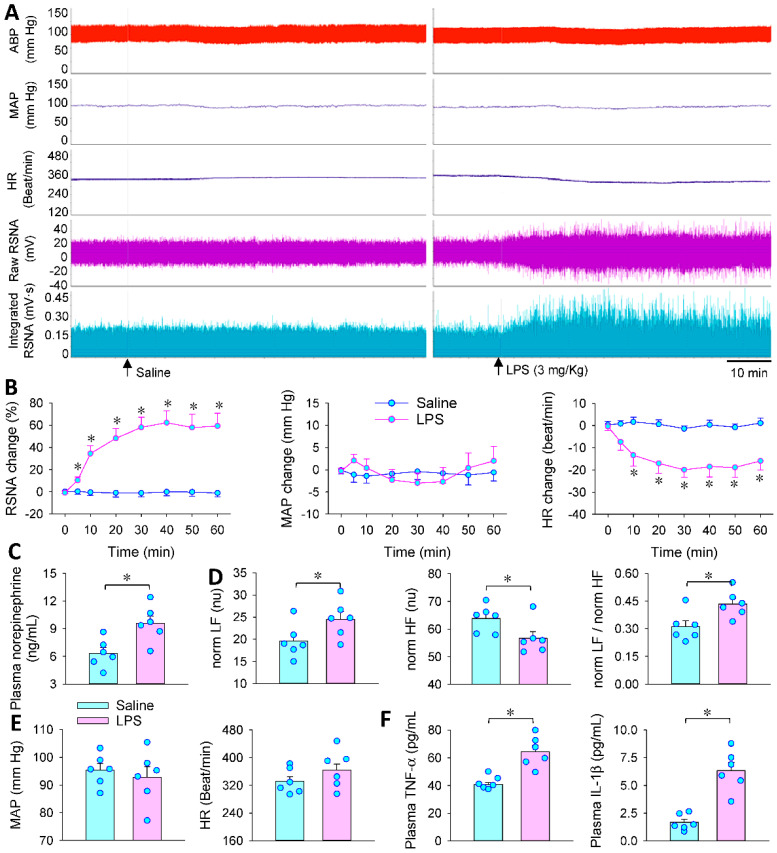
Effects of lipopolysaccharide (LPS) on RSNA, MAP and HR in rats. Rat was subjected to intraperitoneal injection of LPS (3 mg/Kg body weight). (**A**,**B**) LPS-induced changes in renal sympathetic nerve activity (RSNA), mean arterial pressure (MAP) and heart rate (HR) in the first hour after the LPS injection; (**C**–**E**) LPS-induced changes in plasma norepinephrine level, heart rate variability analyses, MAP and HR 24 h after the LPS injection; (**F**) LPS-induced changes in plasma TNF-α and IL-1β levels 24 h after the LPS injection * *p* < 0.05. *n* = 6.

LPS-treated animals are widely used as animal models for sepsis [27,28,29]. LPS induces α2A receptor upregulation in cardiac fibroblasts of mice [35] and can cross the blood-brain barrier to affect brain function [36]. Thus, intraperitoneal injection of LPS was used as a sepsis model to investigate the central role of DEX in attenuating excessive sympathetic activation, oxidative stress and inflammation in sepsis. LPS caused an immediate increase in RSNA and reduction in HR, reaching its maximal effects at approximately 30–40 min (Figure 1A). The bradycardia may be attributed to inflammatory stress and myocardial injury or inhibition due to a sudden increase of LPS. However, no significant change in MAP was observed during the continuous 1-h recordings after LPS injection (Figure 1B). A reasonable explanation may be that the effects of bradycardia on blood pressure counteracts the effects of sympathetic overactivity on blood pressure. Then, measurements were carried out 24 h after LPS injection. LPS increased plasma norepinephrine level (Figure 1C), norm LF and norm LF/norm HF, but reduced norm HF according to heart rate variability analyses (Figure 1D), suggesting that LPS induces long-term sympathetic activation. However, LPS only caused a tendency to reduce MAP and increase HR and the changes did not reach significant levels. The trend of heart rate increase may be attributed to the attenuated myocardial injury or inhibition and the increased sympathetic activity (Figure 1E). Furthermore, LPS increased plasma TNF-α and IL-1β levels. These results indicate that LPS treatment effectively mimics the changes in sympathetic over-activation and inflammation in sepsis. Acute experiments to investigate the effects of DEX in the following studies were carried out 24 h after LPS injection.

### 3.2. Effects of PVN Microinjection of DEX on LPS-Induced RSNA, MAP and HR Changes

**Figure 2:**
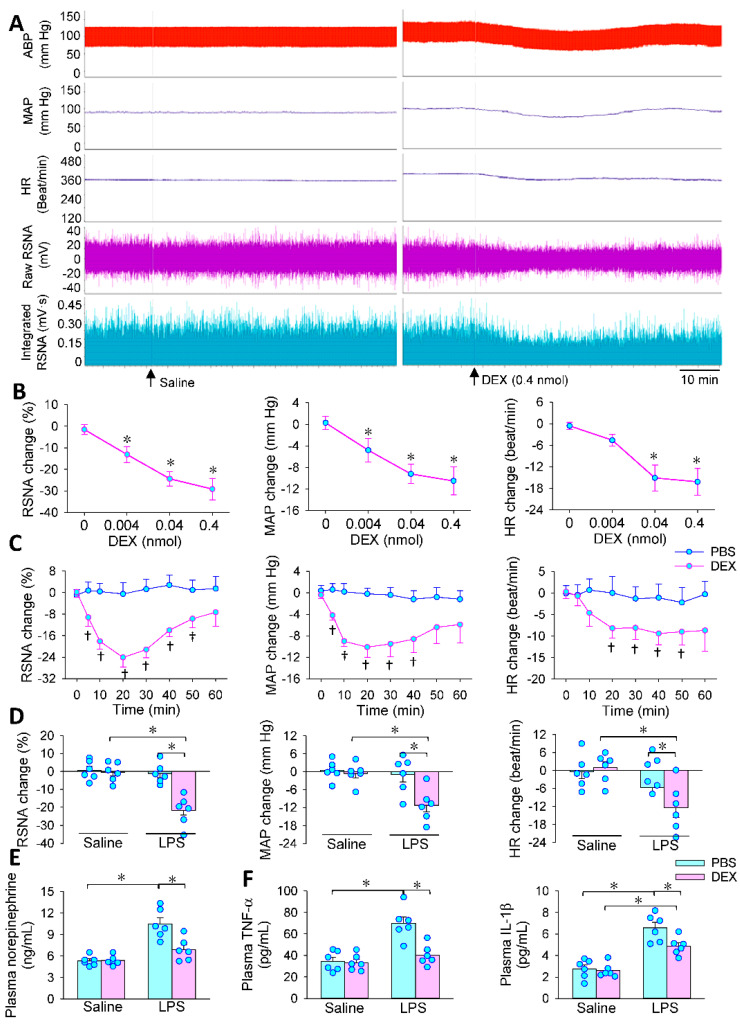
Roles of the DEX microinjection to the paraventricular nucleus (PVN) on LPS-induced RSNA, MAP and HR changes. The PVN microinjection was carried out 24 h after the intraperitoneal injection of LPS. (**A**) Representative images showing the roles of DEX (0.4 nmol) on LPS-induced RSNA, MAP and HR changes; (**B**) dose-effects of DEX (0, 0.004, 0.04 and 0.4 nmol) (* *p* < 0.05 vs. 0 nmol); (**C**) time-effects of DEX († *p* < 0.05 vs. PBS); (**D**) effects of PVN microinjection of DEX (0.4 nmol) on LPS-induced RSNA, MAP and HR changes; (**E**) effects of DEX (0.4 nmol) on LPS-induced changes in norepinephrine level; (**F**) effects of DEX (0.4 nmol) on LPS-induced changes in plasma TNF-α and IL-1β levels (* *p* < 0.05. *n* = 6).

Bilateral PVN microinjection of α2R agonist DEX caused reductions in RSNA, MAP and HR in LPS-treated rats (Figure 2A). DEX in the PVN dose-related reduced the RSNA, MAP and HR, and 0.4 nmol of DEX, which was used in the following studies, was close to its maximal effects (Figure 2B). The effects of DEX lasted at least 50 min, and almost reached its maximal effects at approximately 20–30 min in LPS-treated rats (Figure 2C). However, DEX failed to affect RSNA, MAP and HR in saline-treated rats (Figure 2D). These results indicate that activating α2R with DEX in the PVN attenuates LPS-induced sympathetic activity, which was further confirmed by the reduced plasma norepinephrine levels in DEX-treated rats (Figure 2E). The bradycardia and blood pressure reduction caused by DEX may primarily be secondary to the inhibitory effects of DEX on sympathetic activity, and the bradycardia may further contribute to the reduction in blood pressure. Furthermore, DEX reduced LPS-induced increases in plasma TNF-α and IL-1β levels (Figure 2F), suggesting that PVN microinjection of DEX plays a beneficial role in attenuating LPS-induced inflammation.

### 3.3. α2R in the PVN Mediates the Effects of PVN Microinjection of DEX

**Figure 3:**
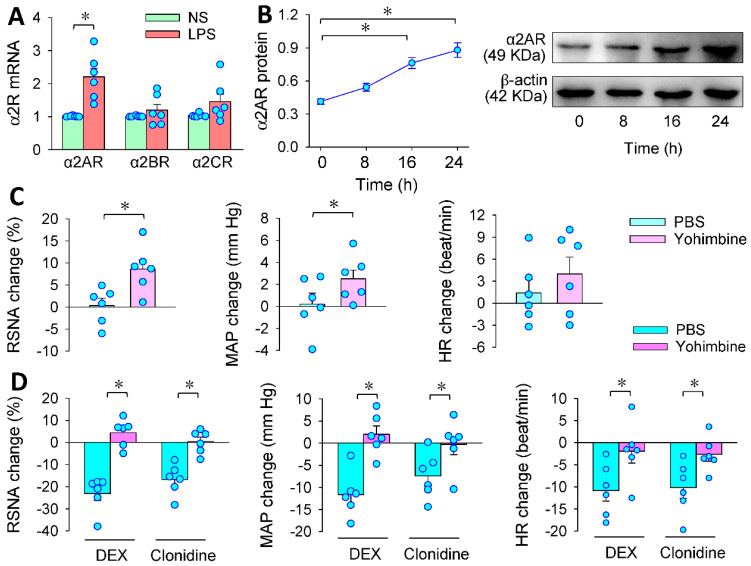
Roles of α2 receptors (α2R) in the effects of DEX in LPS-treated rats. PVN microinjection was carried out 24 h after the intraperitoneal injection of LPS. (**A**) α2R mRNA expressions in the PVN in NS- and LPS-treated rats; (**B**) time-effect of LPS on α2A receptor protein expression in the PVN; (**C**) PVN microinjection of α2 receptors antagonist yohimbine (10 nmol) on RSNA, MAP and HR in LPS-treated rats; (**D**) effects of pretreatment with yohimbine (10 nmol) on the roles of α2R agonist DEX (0.4 nmol) or clonidine (20 nmol) in the PVN in LPS-treated rats. The pretreatment was made 10 min before administration of DEX or clonidine. * *p* < 0.05. *n* = 6.

Presynaptic α2R exists in the PVN [24], and consists of three distinct subtypes, designated as α2AR, α2BR and α2CR [37]. The following experiments were designed to determine which receptor is involved in the effects of DEX. LPS increased α2AR mRNA expression in the PVN but not α2BR or α2CR mRNA expression (Figure 3A). Thus, we further examined α2A protein expression in the PVN in LPS-treated rats. LPS time-related increased the α2AR protein expression in the PVN, and significant α2AR upregulation was found at 16 and 24 h after application of LPS (Figure 3B). Microinjection of α2R antagonist yohimbine into the PVN not only increased RSNA and MAP (Figure 3C), but also completely prevented the effects of DEX on RSNA, MAP and HR in LPS-treated rats. To confirm the roles of α2R in the PVN, the effects of another α2R agonist clonidine were examined. Microinjection of clonidine into the PVN caused similar effects to DEX, which was also abolished by yohimbine (Figure 3D). These results indicate that the effects of DEX are indeed mediated by the activation of presynaptic α2R in the PVN. The upregulation of α2R in the PVN caused by LPS may be a compensatory protective mechanism for attenuating the sympathetic overactivity. However, the descending signaling pathway of DEX-caused α2R activation is not known.

### 3.4. Superoxide in the PVN Mediates the Effects of PVN Microinjection of DEX

**Figure 4:**
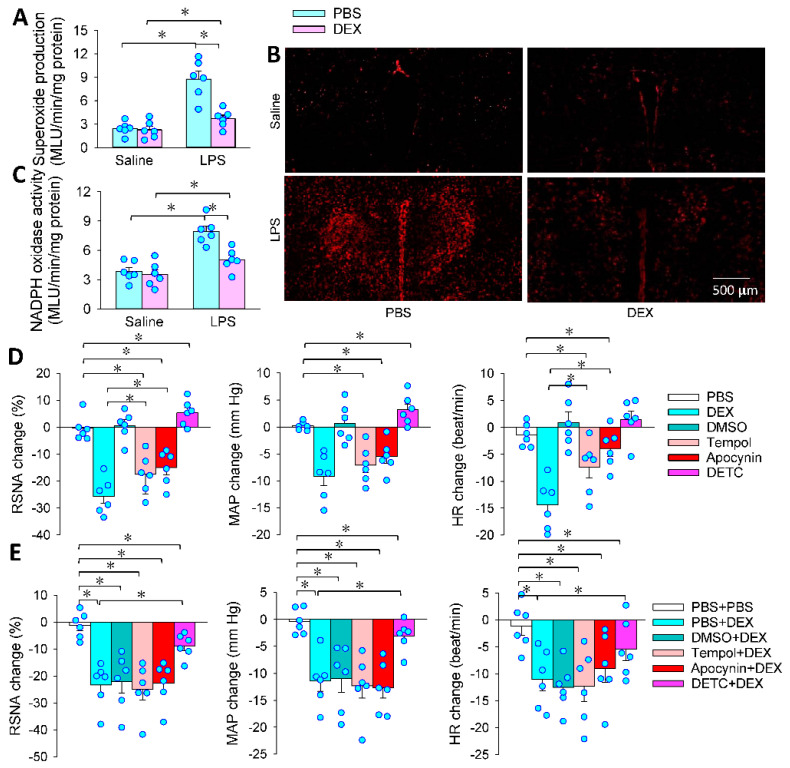
ROS mediates the effects of DEX in LPS-treated rats. PVN microinjection was carried out 24 h after the intraperitoneal injection of LPS. (**A**) Superoxide production in the PVN in saline- and LPS-treated rats; (**B**) DHE fluorescence staining in the PVN in saline- and LPS-treated rats; (**C**) NADPH oxidase activity in saline- and LPS-treated rats; (**D**) effects of superoxide scavenger tempol (20 nmol), NADPH oxidase inhibitor apocynin (1 nmol) and superoxide dismutase inhibitor DETC (10 nmol) in the PVN in LPS-treated rats; (**E**) effects of pretreatment with tempol, apocynin or DETC on the roles of DEX in the PVN in the LPS-treated rats. * *p* < 0.05. *n* = 6.

The NADPH oxidase-derived superoxide production in the PVN promotes sympathetic activation in hypertension [16]. Septic rats with neonatal LPS exposure had higher c-Fos expression in the PVN [38]. It is interesting to determine whether the superoxide production in the PVN may mediate the effects of DEX. We found that LPS increased superoxide production in the PVN, which was abolished by the DEX treatment (Figure 4A). Similar results were further confirmed by DHE fluorescence staining (Figure 4B). Furthermore, LPS increased NADPH oxidase activity in the PVN, which was also attenuated by DEX (Figure 4C). Either tempol (a superoxide scavenger) or apocynin (an NADPH oxidase inhibitor) reduced RSNA, MAP and HR, while DETC (a superoxide dismutase inhibitor) increased RSNA and MAP. The roles of DEX in inhibiting RSNA and MAP were greater than those of tempol and apocynin alone (Figure 4D). Neither tempol nor apocynin enhanced the inhibitory effects of DEX, but DETC attenuated the effects of DEX (Figure 4E). These findings indicate that LPS promoted, while DEX inhibited, the NADPH oxidase-derived superoxide production in the PVN. Inhibition of NADPH oxidase-derived superoxide production might be only one of the mechanisms of DEX in attenuating sympathetic activity.

### 3.5. Intracellular cAMP Signaling Contributes to the Effects of PVN Microinjection of DEX

**Figure 5:**
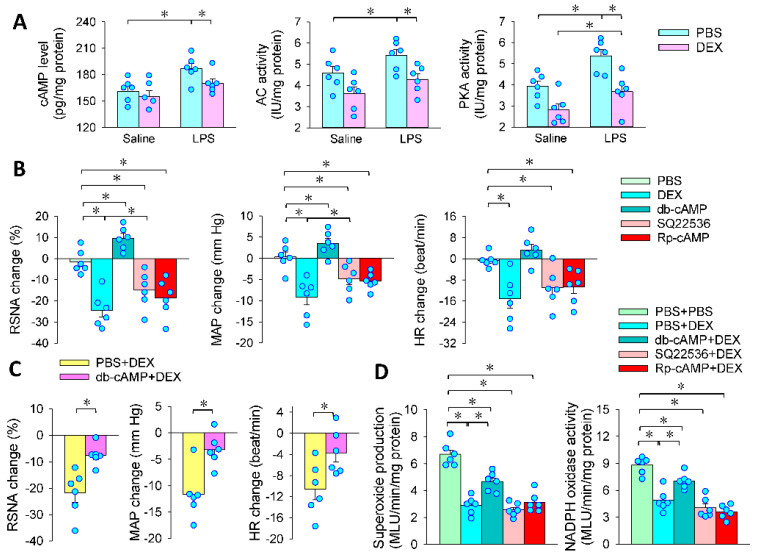
The cAMP-PKA pathway contributes to the effects of DEX in LPS-treated rats. PVN microinjection was carried out 24 h after the intraperitoneal injection of LPS. (**A**) effects of DEX on cAMP level, adenylate cyclase (AC) and protein kinase A (PKA) activity in Saline- and LPS-treated rats; (**B**) effects of PVN microinjection of db-cAMP (a cell permeable cAMP analog, 1 nmol), SQ22536 (an adenylyl cyclase inhibitor, 2 nmol) or Rp-cAMP (a PKA inhibitor, 1 nmol) in LPS-treated rats; (**C**) pretreatment with db-cAMP on the roles of DEX in LPS-treated rats; (**D**) effects of pretreatment with db-cAMP, SQ22536 or Rp-cAMP on the roles of DEX in LPS-treated rats. The pretreatment was carried out 10 min before administration of DEX. * *p* < 0.05. *n* = 6.

α2AR is coupled with the Gi/o member of the G protein family, by which it inhibits adenylate cyclase and thereby reduces intracellular cAMP levels [39,40,41]. Activation of the cAMP-PKA pathway in the PVN increased RSNA and MAP [42]. Therefore, we further examined the cAMP-PKA signaling pathway in mediating the effects of LPS and DEX. LPS increased cAMP level, adenylate cyclase activity and PKA activity, which were prevented by microinjection of DEX in the PVN (Figure 5A). Cell permeable cAMP analog db-cAMP increased the RSNA and MAP. Either SQ22536 (an adenylate cyclase inhibitor) or Rp-cAMP (a PKA inhibitor) had similar effects as DEX in inhibiting RSNA, MAP and HR in LPS-treated rats (Figure 5B). Microinjection of db-cAMP attenuated the roles of DEX in inhibiting RSNA, MAP and HR in LPS-treated rats (Figure 5C). An interesting question is whether the cAMP-PKA signaling pathway is involved in the roles of DEX in attenuating superoxide production in the PVN. Microinjection of db-cAMP attenuated the roles of DEX, but SQ22536 or Rp-cAMP did not further enhance the effects of DEX in inhibiting superoxide production and NADPH oxidase activity in LPS-treated rats (Figure 5D). The results indicate that the cAMP-PKA pathway is partially responsible for the effects of DEX in inhibiting NADPH-derived superoxide production and sympathetic activation in LPS-induced sepsis. There might be some other signaling pathways involved in the effects of DEX.

### 3.6. GABA in the Effects of DEX in PVN

**Figure 6:**
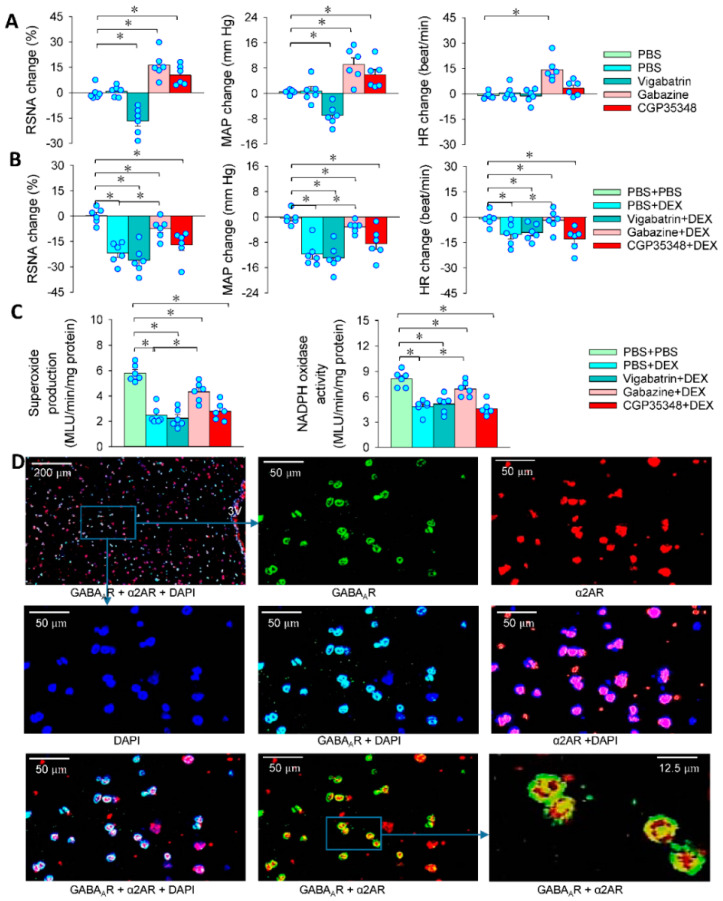
GABA in the effects of DEX in the LPS-treated rats. PVN microinjection was carried out 24 h after the intraperitoneal injection of LPS. (**A**) Effects of PVN microinjection of vigabatrin (a GABA transaminase enzyme inhibitor, 10 nmol), gabazine (a GABA_A_ receptor antagonist, 0.1 nmol) or CGP35348 (a GABA_B_ receptor antagonist, 10 nmol) on RSNA, MAP and HR; (**B**) effects of pretreatment with vigabatrin, gabazine or CGP35348 on the roles of DEX in reducing RSNA, MAP and HR in LPS-treated rats; (**C**) effects of pretreatment with vigabatrin, gabazine or CGP35348 on the roles of DEX in superoxide production and NADPH oxidase activity in LPS-treated rats (pretreatment was carried out 10 min before administration of DEX); (**D**) immunofluorescent staining for α2A receptors (red), GABA_A_ receptors (green) in the PVN. Nuclei were stained with DAPI (blue). Abbreviation: 3V, the third ventricle. * *p* < 0.05. *n* = 6.

It is known that GABA in the PVN plays an important role in inhibiting sympathetic outflow [43]. DEX rapidly promotes GABA release in the dorsal motor nucleus of the vagus [44]. The α2AR and GABA immunoreactivities were co-localized in a close position in the PVN neurons [45]. It is interesting to determine whether GABA contributes to the roles of DEX in LPS-treated rats. Inhibition of GABA transaminase with vigabatrin to increase the GABA concentration in the PVN reduced RSNA and MAP, while a blockade of GABA_A_ receptor blocker gabazine or a GABA_B_ receptor blocker CGP35348 increased RSNA and MAP (Figure 6A). Vigabatrin did not potentiate the roles of DEX in inhibiting RSNA, MAP and HR. Gabazine attenuated the roles of DEX, but CGP35348 had no significant effects on the roles of DEX in inhibiting RSNA, MAP and HR (Figure 6B). Moreover, gabazine attenuated, but vigabatrin and CGP35348 had no significant effects on the roles of DEX in inhibiting the superoxide production and NADPH oxidase activity (Figure 6C). Immunofluorescent staining showed that both α2AR and GABA_A_ receptors were expressed in the PVN (Figure 6D). The findings indicate that DEX attenuates NADPH oxidase-mediated superoxide production and sympathetic activation, which are partially mediated by promoting GABA release and GABA_A_ receptor activation.

### 3.7. PVN in the Roles of Intravenous DEX in Inhibiting Sympathetic Activation

**Figure 7:**
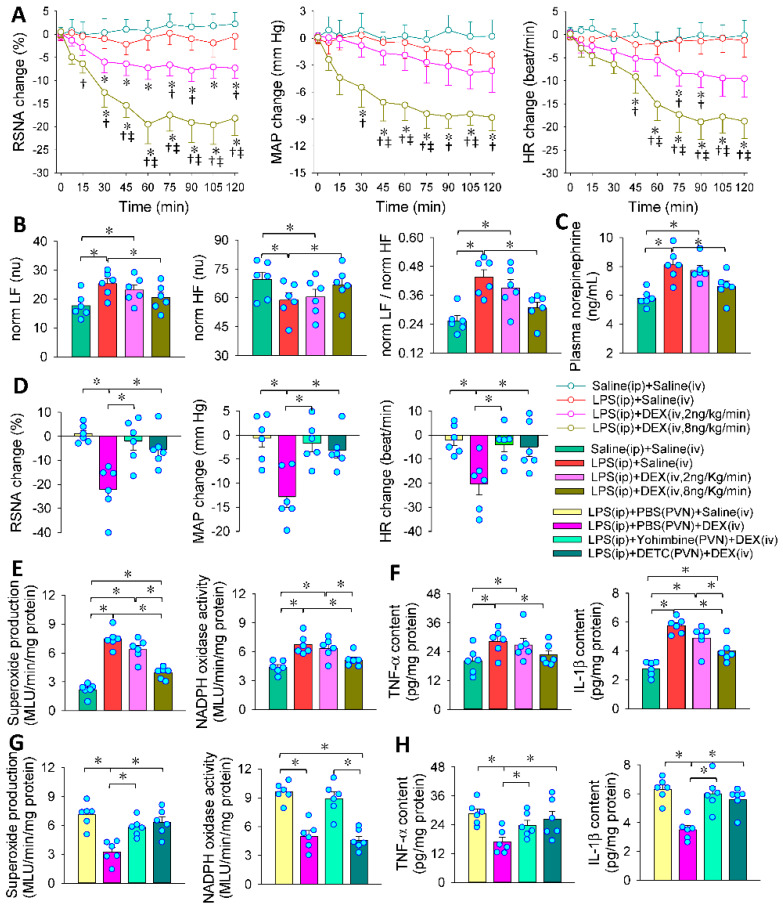
Roles of the PVN in the effects of intravenous infusion (iv) of DEX on the LPS-induced sympathetic activation. Intravenous infusion of DEX for 4 h was carried out 24 h after intraperitoneal injection (ip) of LPS. Bilateral PVN microinjection was performed 5 min before the infusion of DEX. (**A**) Effects of DEX (2 or 8 ng/Kg/min, iv) on the LPS-induced RSNA, MAP and HR changes (* *p* < 0.05 vs. Saline+Saline) († *p* < 0.05 vs. LPS+Saline) (‡ *p* < 0.05 vs. LPS+DEX) (2 ng/Kg/min); (**B**,**C**) effects of DEX (2 or 8 ng/Kg/min, iv) on the LPS-induced heart rate variability changes and plasma norepinephrine level changes; (**D**) effects of PVN microinjection of yohimbine (10 nmol) or DETC (10 nmol) on the roles of DEX (8 ng/Kg/min, iv) in inhibiting LPS-induced RSNA, MAP and HR changes; (**E**,**F**) effects of intravenous infusion (iv) of DEX on the roles of the LPS-induced changes in oxidative stress and inflammation in the PVN; (**G**,**H**) effects of microinjection of yohimbine (10 nmol) or DETC (10 nmol) in the PVN on the roles of DEX in inhibiting LPS-induced changes in oxidative stress and inflammation in the PVN (* *p* < 0.05. *n* = 6).

We are interested in knowing if the PVN contributes to the roles of DEX in LPS-induced sympathetic overactivity. The dosage of intravenous infusion of DEX (2 or 8 ng/Kg/min) was selected according to the dose range of sedative effect of DEX commonly used in patients. Intravenous infusion of a high dose of DEX persistently reduced RSNA, MAP and HR in LPS-treated rats, reaching its maximal effects at approximately 60 min (Figure 7A). The inhibitory effects of DEX on LPS-induced sympathetic activation in LPS-treated rats were further confirmed by the changes in the heart rate variability (Figure 7B) and plasma norepinephrine levels (Figure 7C). Microinjection of yohimbine to block α2R or DETC to increase superoxide levels in the PVN almost abolished the roles of intravenous administration of DEX in inhibiting LPS-induced changes in RSNA, MAP and HR (Figure 7D). It is known that oxidative stress and inflammation in the PVN increase sympathetic outflow [16,46]. The present study has shown that NADPH oxidase-derived superoxide production in the PVN mediates the inhibitory effects of DEX on sympathetic activity as we described above. We further found that intraperitoneal injection of LPS increased the superoxide production, NADPH oxidase activity, TNF-α and IL-1β levels in the PVN, and the effects were inhibited by intravenous injection of DEX (Figure 7E,F). Most importantly, the PVN microinjection of α2R antagonist yohimbine or superoxide dismutase inhibitor DETC almost abolished the roles of DEX in inhibiting LPS-induced oxidative stress and inflammation in the PVN in LPS-treated rats (Figure 7G,H). These findings indicate that systemic administration of DEX attenuates sympathetic activation in LPS-induced sepsis primarily by acting on α2R in the PVN and resulting in subsequent attenuation of oxidative stress and inflammation in the PVN.

### 3.8. PVN in the Roles of Intravenous DEX in Attenuating Systemic Oxidative Stress, Inflammation and Organ Injury

**Figure 8:**
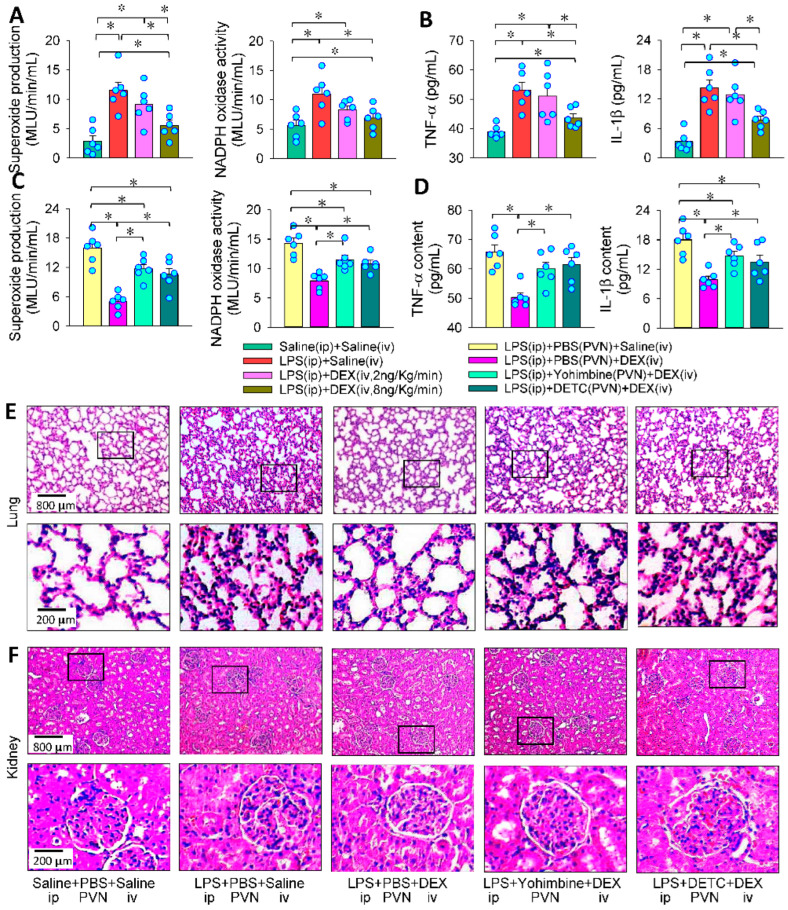
Roles of the PVN in the effects of intravenous infusion (iv) of DEX on the LPS-induced oxidative stress and inflammation. Intravenous infusion of DEX for 4 h was carried out 24 h after intraperitoneal injection (ip) of LPS. Bilateral PVN microinjection was performed 5 min before the infusion of DEX. (**A**,**B**) Effects of DEX (2 or 8 ng/Kg/min) on the LPS-induced changes in plasma superoxide production, NADPH oxidase activity, TNF-α and IL-1β level; (**C**,**D**), effects of PVN microinjection of yohimbine (10 nmol) or DETC (10 nmol) on the roles of DEX in inhibiting LPS-induced changes in plasma superoxide production, NADPH oxidase activity, TNF-α and IL-1β levels; (**E**,**F**) HE staining showed the effects of PVN microinjection of yohimbine (10 nmol) or DETC (10 nmol) on the roles of DEX in attenuating LPS-induced changes in lung and kidney injury. * *p* < 0.05. *n* = 6.

Inflammation and oxidative stress contribute to the pathogenesis of sepsis [47]. DEX exerts powerful roles in anti-inflammation, anti-oxidative stress, sympatholytic and organ-protection effects [48]. The α2R agonists DEX and clonidine can improve the survival rate in septic animal models, which is supposed to be related to the sympatholytic effect that reduces pro-inflammatory mediators [13]. However, it is still unknown whether the central sympatholytic effect of DEX could play important roles in systemic anti-inflammation, anti-oxidative stress and organ-protection in sepsis. Intravenous injection of DEX reduced plasma superoxide production, NADPH oxidase activity, TNF-α and IL-1β levels (Figure 8A,B), which were partially counteracted by the PVN microinjection of yohimbine or DETC (Figure 8C,D). LPS reduced the alveolar cavity and increased alveolar wall thickness and inflammatory cell infiltration in the lungs, which were attenuated by intravenous infusion of DEX. The effects of DEX were weakened by PVN microinjection of yohimbine or DETC (Figure 8E). Furthermore, intravenous infusion of DEX attenuated the LPS-induced glomerular deformation, interstitial tubule injury and inflammatory cell infiltration in kidneys, which were partially prevented by PVN microinjection of yohimbine or DETC (Figure 8F). The results indicate that the roles of intravenous administration of DEX are partially dependent on its central effects by activating α2R and reducing its downstream superoxide and pro-inflammatory factor production, causing excessive sympathetic activity.

## 4. Discussion

**Figure 9:**
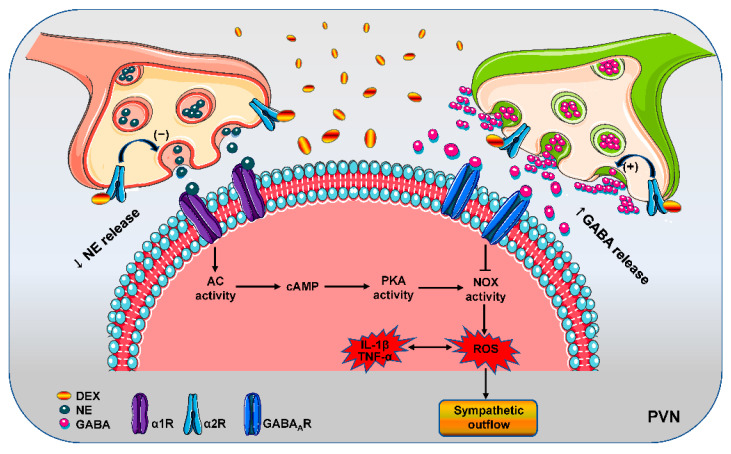
Schematic diagram showing the roles of DEX in the PVN in regulating sympathetic activity. Abbreviations: AC, adenylate cyclase; DEX, dexmedetomidine; GABA_A_R, type A γ-aminobutyric acid receptor; IL-1β, interleukin-1β; NE, norepinephrine; NOX, NADPH oxidase; PKA, protein kinase A; PVN, paraventricular nucleus; ROS, reactive oxygen species; TNF-α, tumor necrosis factor-α; α1R, α1 adrenergic receptor; α2R, α2 adrenergic receptor.

Excessive sympathetic activity contributes to inflammation and dysfunction of multiple organs in sepsis [7]. The α2R agonist DEX is used in the intensive care management of patients and plays important roles in anti-inflammation, anti-oxidative stress, sympatholytic effect and organ-protection [48]. Little is known about the contribution of the central sympatholytic effect of DEX to systemic inflammation, oxidative stress and organ-protection. The main novel findings in this study are that activating α2R with microinjection of DEX to the PVN not only inhibits adenosine cyclase-cAMP-PKA signaling, but also promotes GABA release and GABA_A_ receptor activation, both of which reduce NADPH oxidase-dependent superoxide production in the PVN, and then prevent sympathetic overactivity in LPS-induced rats. Intravenous infusion of DEX inhibits NADPH oxidase-dependent superoxide production by activating α2R in the PVN, and then prevents LPS-induced sympathetic activation, which at least partially contributes to its anti-inflammatory, anti-oxidative stress and organ-protective effects in septic rats (Figure 9). We speculate that DEX can attenuates sepsis by reducing sympathetic activity, oxidative stress, inflammation and organ damage.

Sympathetic activity was augmented in septic animals and patients [4]. The enhanced sympathetic outflow in sepsis contributes to inflammation and organ dysfunction [7,8,9]. LPS-induced sympathetic activation was prevented by microinjection of the selective α2R agonist DEX in the PVN. Another α2R agonist clonidine in the PVN had similar effects to DEX. The effects of DEX or clonidine were completely prevented by the α2R antagonist yohimbine. Moreover, LPS increased α2AR expression in the PVN. The results indicate that microinjection of DEX into the PVN reduced sympathetic activity by acting on α2R. The upregulation of α2R in LPS-treated rats may be a compensatory mechanism for counteracting LPS-induced sympathetic overactivity.

LPS increases the intracellular cAMP level in human gingival fibroblasts and neuroendocrine cell line PC12 [49,50]. LPS recognizes TLR4, and thus activates the transcription of TNF-α and IL-1β [51] and the cAMP signaling pathway [52,53]. The upregulation of cAMP in the PVN increases sympathetic outflow [42]. LPS increased cAMP level, adenylate cyclase and PKA activities in the PVN, which were abolished by microinjection of DEX to the PVN. Inhibition of adenylate cyclase or PKA had a similar role to DEX in inhibiting RSNA, while cAMP analog increased RSNA and partially counteracted the role of DEX in inhibiting RSNA in LPS-treated rats. The results indicate that LPS-induced sympathetic overactivity is partially mediated by activating the cAMP-PKA signaling pathway in PVN, and the DEX in the PVN inhibits adenylate cyclase activity and cAMP-PKA signaling, and thereby attenuates sympathetic activity in LPS-induced rats. It is well known that activation of presynaptic α2R reduces norepinephrine release [26], which binds to postsynaptic receptors to activate the cAMP-PKA pathway [54]. The α1R antagonist prazosin abolishes the roles of norepinephrine in increasing intracellular cAMP levels in slices from the cerebral cortex [55]. The role of DEX in inhibiting adenylate cyclase activation is attributed to the reduced presynaptic norepinephrine release in the PVN. However, the changes in cAMP-PKA signaling in the PVN cannot fully explain the effects of LPS and DEX; there must be other signaling involved in their effects.

GABA in the PVN inhibits sympathetic activity [43]. Systemic injection of LPS activates microglia in the PVN, and thereby promotes proinflammatory cytokine production in the PVN. The proinflammatory cytokines promote the excitation of preautonomic parvocellular PVN neurons and decrease GABA release to the PVN neurons, leading to excessive sympathetic activity [56]. In the present study, LPS increased TNF-α and IL-1β levels in the PVN, and the effects were attenuated by microinjection of DEX to the PVN. Upregulation of GABA in the PVN was reduced, while blockades of GABA_A_ or GABA_B_ receptors increased sympathetic outflow in LPS-treated rats. The roles of DEX in inhibiting LPS-induced sympathetic overactivity were attenuated by the blockade of GABA_A_ receptors in the PVN. Both α2AR and GABA receptors were expressed in the PVN. These results indicate that DEX promotes GABA release, and that the released GABA inhibits sympathetic activity by GABA_A_ receptors.

Superoxide production in the PVN promotes sympathetic activation [16]. PVN microinjection of DEX abolished LPS-induced NADPH oxidase activity and superoxide production in the PVN. Either the superoxide scavenger tempol or the NADPH oxidase inhibitor apocynin reduced sympathetic outflow. The superoxide dismutase inhibitor DETC increased sympathetic outflow and attenuated the roles of DEX in inhibiting sympathetic activity. These findings indicate that LPS promoted, while DEX inhibited, NADPH oxidase-derived superoxide production in the PVN. Microinjection of the cAMP analog db-cAMP or the GABA_A_ receptor antagonist gabazine attenuated the roles of DEX in inhibiting NADPH oxidase-derived superoxide production in the PVN in LPS-treated rats. These results indicate that both the cAMP-PKA signaling pathway and GABA-GABA_A_ receptor signaling mediate the effects of DEX in inhibiting NADPH-derived superoxide production and sympathetic activation in LPS-induced sepsis. The findings were supported by previous findings that cAMP-PKA signaling contributes to ROS production [57,58], and that GABA attenuates oxidative stress [59,60,61].

DEX is used in the intensive care management of patients and is conducive to organ protection of patients with sepsis [22,23]. It is still unknown whether the beneficial roles of systemic administration of DEX are mainly dependent upon its central action to inhibit the sympathetic overactivity in sepsis. We found that intravenous infusion of DEX attenuated sympathetic outflow to the kidney and plasma norepinephrine level in LPS-induced sepsis. Heart rate variability analyses showed that DEX attenuated the sympathetic–parasympathetic imbalance by inhibiting sympathetic activity and increasing parasympathetic activity in the LPS-treated rats. The inhibitory effects of DEX are almost abolished by the PVN microinjection of yohimbine to block α2R or DETC to increase the superoxide content in the PVN. The production of superoxide and inflammatory cytokines in the PVN leads to excessive sympathetic activity in hypertension [16]. LPS increased NADPH oxidase-dependent superoxide production and inflammatory cytokine release in the PVN, which were almost prevented by the PVN microinjection of yohimbine and DETC. These findings indicate that the effects of intravenous infusion of DEX in preventing the LPS-induced sympathetic activation are primarily mediated by its action on the α2R to inhibit superoxide and inflammatory cytokine production in the PVN, which is supported by the fact that DEX can cross blood-brain barrier easily [25].

It is well known that systemic administration of DEX plays beneficial roles in anti-inflammation, anti-oxidative stress and organ-protection [48]. Excessive sympathetic activity contributes to inflammation and organ dysfunction in sepsis [7,8,9,10]. Central blockage of sympathetic outflow attenuated inflammation and prolonged survival in a septic animal model [13]. We found that the effects of intravenous injection of DEX in attenuating LPS-induced systemic oxidative stress and inflammation were attenuated by PVN microinjection of yohimbine or DETC. These results indicate that the anti-inflammation and anti-oxidative stress effects of intravenous injection of DEX are partially mediated by its action on α2R in the PVN to inhibit sympathetic outflow. Moreover, the effects of DEX in attenuating LPS-induced lung and kidney injury were attenuated by PVN microinjection of yohimbine or DETC. The organ-protection effect of DEX is attributed to both peripheral anti-inflammation and anti-oxidative stress effects and central sympatholytic effects. The latter not only attenuates inflammation and oxidative stress, but also improves the blood supply to organs. It is noted that the effects of two doses of intravenous infusion of DEX were examined in the rats, which correspond to the upper and lower limits of the sedative dose of DEX commonly used in patients calculated per kilogram of body weight. The high dose showed stronger effects, but the low dose showed only mild effects, suggesting that the selection of adequate doses of DEX is necessary for the treatment. The limitation of this study is that the effects of DEX were investigated in early and mild/moderate LPS-induced sepsis. The findings may not necessarily be applicable to late and more severe sepsis.

There has been conflicting evidence about the positive effects of DEX in the setting of sepsis in humans. A randomized clinical trial showed that there was no significant improvement in mortality and ventilator free days in septic patients with DEX compared with sedatives without DEX [62]. There was no significant difference in outcomes between the septic adults treated with DEX and those treated with propofol [63]. However, a recent meta-analysis of 19 randomized controlled trials that enrolled 1929 patients showed the benefit of DEX in septic patients [64]. Future randomized controlled trials need to identify the population of patients with sepsis who can benefit most from DEX and its optimal regimen. It is worth noting that the beneficial effects of DEX on sepsis were obtained from an LPS-induced rat septic model, which may not be completely applicable to human sepsis.

## 5. Conclusions

Microinjection of DEX in the PVN attenuates LPS-induced sympathetic overactivity via α2R-mediated adenosine cyclase inhibition and GABA release in rats. The former reduces cAMP level and PKA activity, and the latter activates GABA_A_ receptors. Both of them cause inhibition in NADPH oxidase activity and a subsequent decrease in superoxide production in the PVN. Eventually, the role of DEX in preventing the LPS-induced superoxide production is attributed to the normalization of sympathetic outflow in LPS-induced sepsis. Intravenous infusion of DEX abolishes the LPS-induced sympathetic overactivity, which is primarily mediated by α2R in the PVN in rats. The normalization of sympathetic outflow caused by the intravenous infusion of DEX plays a vital role in attenuating inflammation, oxidative stress and organ injury in the LPS-induced sepsis. Inhibition of excessive sympathetic activation might be an important therapeutic strategy in sepsis.

## Data Availability

The data are contained within the article and Supplementary Materials.

## Supplementary Materials

The following supporting information can be downloaded at https://www.mdpi.com/article/10.3390/antiox11122395/s1: Table S1: Primers for RT-PCR analysis in rats.
