# Supplementary material for: Dexmedetomidine Attenuates Lipopolysaccharide-Induced Sympathetic Activation and Sepsis via Suppressing Superoxide Signaling in Paraventricular Nucleus
Source: Antioxidants (Basel). 2022 Dec 2;11(12):2395. doi: 10.3390/antiox11122395 (PMC9774688; doi:10.3390/antiox11122395)
Supplement: Supplementary file 1 [file antioxidants-11-02395-s001.zip › antioxidants-1969735-supplementary.pdf]

Table S1 Primers for RT-PCR analysis in rats

Name	Primer	Sequence	Accession no.
Rat α2A receptor	Forward	5'- GCACCACTTGCCCCAGTAA -3'	NM_012739
	Reverse	5'- AGCACACCCACCCGTTCTTCTT -3'	
Rat α2B receptor	Forward	5'- GATATAACCAGTTCCACCCCAGCTCAGAA -3'	NM_138505
	Reverse	5'- AGTTGGGAAGACAACCAGGAGGTAGA -3'	
Rat α2C receptor	Forward	5'- CGCGAGAAACGCTTCACCTT -3'	NM_138506
	Reverse	5'- CACGGCAGATGCCATACAGG -3'	
Rat GAPDH	Forward	5'- TTCCAGGAGCGAGATCCCGCTAAC -3'	NM_017008
	Reverse	5'- TTCAGGTGAGCCCCAGCCTTCT -3'	
